# Evaluation of an RNAseq-Based Immunogenomic Liquid Biopsy Approach in Early-Stage Prostate Cancer

**DOI:** 10.3390/cells10102567

**Published:** 2021-09-28

**Authors:** Leander Van Neste, Kirk J. Wojno, Ricardo Henao, Shrikant Mane, Howard Korman, Jason Hafron, Kenneth Kernen, Rima Tinawi-Aljundi, Mathew Putzi, Amin I. Kassis, Philip W. Kantoff

**Affiliations:** 1 Immunis.AI, Royal Oak, MI 48067, USA; amin_kassis@hms.harvard.edu; 2 Infinia ML, Duke University, Durham, NC 27708, USA; ricardo.henao@duke.edu; 3 Duke University, Durham, NC 27708, USA; 4 Yale University Center for Genomic Analysis, New Haven, CT 06511, USA; shrikant.mane@yale.edu; 5 Comprehensive Urology Center, Royal Oak, MI 48073, USA; hkorman@urologist.org; 6 School of Medicine, Wayne State University, Detroit, MI 48201, USA; 7 Michigan Institute of Urology, St. Clair Shores, MI 48081, USA; hafronj@michiganurology.com (J.H.); kernenk@michiganurology.com (K.K.); aljundir@michiganurology.com (R.T.-A.); 8 Urology Austin, Austin, TX 78705, USA; matthew.putzi@urologyaustin.com; 9 Harvard Medical School, Harvard University, Boston, MA 02115, USA; 10 Memorial Sloan Kettering Cancer Center, New York, NY 10065, USA; Philip.kantoff@convergentrx.com; 11 Convergent Therapeutics Inc., Cambridge, MA 02138, USA

**Keywords:** cancer, immune, cells, transcriptomics, phagocytosis, CD14+, CD2+, gradient, boosting

## Abstract

The primary objective of this study is to detect biomarkers and develop models that enable the identification of clinically significant prostate cancer and to understand the biologic implications of the genes involved. Peripheral blood samples (1018 patients) were split chronologically into independent training (*n* = 713) and validation (*n* = 305) sets. Whole transcriptome RNA sequencing was performed on isolated phagocytic CD14+ and non-phagocytic CD2+ cells and their gene expression levels were used to develop predictive models that correlate to adverse pathologic features. The immune-transcriptomic model with the highest performance for predicting adverse pathology, based on a subtraction of the log-transformed expression signals of the two cell types, displayed an area under the curve (AUC) of the receiver operating characteristic of 0.70. The addition of biomarkers in combination with traditional clinical risk factors (age, serum prostate-specific antigen (PSA), PSA density, race, digital rectal examination (DRE), and family history) enhanced the AUC to 0.91 and 0.83 for the training and validation sets, respectively. The markers identified by this approach uncovered specific pathway associations relevant to (prostate) cancer biology. Increased phagocytic activity in conjunction with cancer-associated (mis-)regulation is also represented by these markers. Differential gene expression of circulating immune cells gives insight into the cellular immune response to early tumor development and immune surveillance.

## 1. Introduction

Immune cell gene expression changes in response to different conditions or stimuli such as infection, ageing, and diseases, including cancer, have revealed relevant new pathways and co-expression networks [1,2,3,4]. Efficient next-generation RNA sequencing platforms have facilitated the ability to perform whole genome expression profiling of individual populations of immune cells [5,6,7,8] as a novel means of searching for patterns of gene expression to aid in the identification of meaningful signals of various disease states. For example, whole-blood RNA expression has been studied in castration resistant prostate cancer (PCa) by various methods with good results predicting survival as well as suggesting dysregulation of the immune system [9,10,11]. Recent interest in expression profiles of particular leukocyte subsets suggests that diagnostic information for many disorders may be contained therein [12,13,14,15,16,17,18,19,20,21]. Mononuclear phagocytic cells including the various CD14+ subsets have been studied extensively in various disease states including some solid tumors, i.e., lung and pancreas [22,23,24]. This study aims to determine if the differential transcriptomic profiles of CD14+ and CD2+ cell populations are associated with features of adverse pathology in early stage, clinically localized prostate cancer.

Prostate cancer is a relatively slow growing, heterogenous, oligoclonal epithelial malignancy that commonly affects the prostate of men as a result of germline genetic predispositions [25], accumulation of mutations of oncogenes and tumor suppression genes [26,27] and aberrant epigenetic events, as well as immune-evasion [28], and cancer immunoediting [29]. Currently, it is hypothesized that cancer immunoediting occurs in three phases [30]. First, during the elimination phase, tumor cells are killed by natural killer (NK), and CD4+ and CD8+ T-lymphocytes [31]. During the second, equilibrium phase tumor cells that have not been eliminated and that do not elicit an immunogenic response are perpetuated, while (epi)genetic defects progressively accumulate and clonal selection occurs [32]. Finally, the escape phase sets in, during which the immune system is unable to destroy the tumor cells which can grow and expand in an uncontrolled manner, resulting in the appearance of clinically detectable tumors. During these three phases the immune cells are hypothesized to alter their (epi)genomic profile in response to the increasing stress on the immune system that occurs while trying to maintain control of a developing tumor [32]. These changes can be studied in isolated, purified immune cell subpopulations, or by single cell sequencing.

Phagocytosis is one of the main mechanisms of innate immune defense. Macrophages initiate phagocytosis by various receptors (mannose receptors, scavenger receptors, Fc γ receptors, and complement receptors 1, 3, and 4). Macrophages are long-lived and can continue phagocytosis by forming new lysosomes [33,34]. In this paper, we use whole transcriptome sequencing analysis to study how phagocytosis of apoptotic tumor cells affects the transcriptome of macrophages. From each patient, the transcriptome of the CD2+ lymphocytic cells is also sequenced, both as a source of signals of the body’s lymphocytic response to the presence of a developing tumor, but also as a patient-specific white blood cell control for the phagocytic macrophages that potentially possess cancer-specific signals when cleaning up apoptotic tumor cells. To this end, the RNA expression of the CD14+ phagocytic cells in the peripheral blood is normalized for non-phagocytic CD2+ cell expression capturing both aspects of the immune response at the same time. Therefore, it is hypothesized that this CD2-normalized CD14 signal could serve as a valuable metric in predicting features for the presence of aggressive, clinically significant cancer. Relevant gene sets that are differentially expressed between these two cell types of the same patient are thus used to develop models predicting the presence of adverse pathologic features. Pathway and ontology analyses are then used to provide biological insights into the cooperative nature of the two cell types with respect to transcriptomic activation in response to tumor presence. This approach might also be valuable to investigate therapeutic response and minimal residual disease [35].

Prostate cancer was selected as a model system to study this hypothesis due to the high prevalence in the population of early stage, clinically localized tumors that would be available for study. Early-stage prostate cancer can be divided into indolent tumors that are watched by active surveillance versus tumors demonstrating aggressive pathologic features that require early intervention to prevent metastasis and mortality [36,37]. This transition from indolent to aggressive tumor corresponds to the point of escape from the immune system where the transcriptomic immune response signal is likely to be maximal. 

## 2. Materials and Methods

Patient population: Blood samples were collected from 1018 men who were visiting their urologist and were suspected of having prostate cancer or were known to have untreated prostate cancer and signed an informed consent to this IRB approved study (WIRB # 20130028).

Inclusion criteria: Men were eligible for enrollment in the study if they (i) were determined by their physician to have a risk profile that warranted either a prostate biopsy, (ii) had a biopsy > 90 days prior to but <1 year of study entry and had not undergone definitive therapy, (iii) were on active surveillance after the diagnosis of prostate cancer such that a biopsy would be performed within the next year but at least 30 days after the blood draw, or in combination.

Exclusion criteria: Men were not eligible for enrollment in this study if they (i) were less than 40 years of age as prostate cancer in younger men has different behavior and characteristics, (ii) had any known concurrent cancer except non-melanoma skin cancer or any history of cancer in the last 5 years, or (iii) had any form of androgen deprivation therapy (ADT) with the exception of 5-alpha reductase inhibitors.

Clinical and pathological data: Clinical, laboratory, and pathology data of each patient was abstracted from the electronic medical record and entered into an electronic data capture (EDC) system by the research teams at the various institutions under the IRB approved protocol (Comprehensive Urology (CU), Metropolitan Detroit, Michigan; Michigan Institute of Urology (MIU) Metropolitan Detroit, Michigan; and Urology Austin, Austin, Texas). The current gold standard of care (SOC) for prostate cancer detection remains the 12-part transrectal, ultrasound-guided (TRUS) biopsy which was used at each of the institutions during the time period of the study. Standard 12 core systematic biopsies were performed with allowance for additional cores at the urologist’s discretion. Pathologists at all three institutions agreed on the main standard data points to be included in the needle biopsy pathology reports. The current International Society of Urological Pathology (ISUP) modified Gleason grading system was used [38] and the data from the highest-grade group of a single core was recorded. The maximal cross-sectional surface area of tumor on a single core and the number of positive cores were recorded in the EDC. A portion of the cases had pathology review to ensure grading uniformity between sites. For this study, the presence of adverse pathologic features was defined as any (i) Gleason grade group (GG) 4 or 5, (ii) any GG 3 with greater than 3 cores positive or greater than 30% of a core involved, or (iii) GG 2 with greater than 6 cores positive and greater than 60% of a core positive. For patients who had undergone radical prostatectomy, adverse pathology was defined as either GG 4 or GG 5 of any size, GG 3 with >30% of prostate involved, GG 3 with >10 mm tumor size, GG 2 with >60% of prostate involved or GG 2 with >20 mm tumor size. All available data was considered, and the most aggressive pathology was used to define cases as having adverse pathology with patients being followed up to 5 years. The demographic information of the study population is presented in the results section; Supplementary Figure S1 contains details of data integrity, missing values, and imputation.

Sample collection and transport: Blood samples were obtained from the three large urology practices. All enrolled patients signed written informed consent forms per ethical guidelines of the Institutional Review Board. Blood samples were collected in four K2EDTA BD VacutainerTM tubes (Cat. No. 366643, BD Biosciences, San Jose, CA, USA) and transferred to the processing locations on ice at 4 °C and processed 4 h after draw time.

CD2 and CD14 cell separation: Blood was pooled from 3 blood tubes at 4 °C and split into 1/3 and 2/3 aliquots for CD2 and CD14 cell type isolations, respectively. Specially formulated positive selection magnetic-activated cell sorting (MACS) microbeads using anti-CD2 antibodies and anti-CD14 antibodies (Cat. No. 130-101-329 and 130-101-328, respectively, Miltenyi Biotech, Bergisch Gladbach, Germany) were added to the aliquots of blood at a volume of 25 µL CD2 beads per 1 mL blood and 50 µL CD14 beads per 1 mL blood. Beads were incubated with the blood samples for 10 min at 4 °C. The blood-bead suspensions were then processed at 4 °C using a positive selection template on the autoMACS Pro Separator (Miltenyi Biotech) to isolate the CD2 and CD14 cells. Small aliquots of the isolated CD2 and CD14 cells were removed for flow cytometry analysis while the remaining cells were pelleted by a 10-min centrifugation at 300× *g* at 4 °C. Following centrifugation, the supernatant was removed and 700 µL of room temperature QIAzol Lysis Reagent (Cat. No. 79306, Qiagen, Hilden, Germany) was added to each cell pellet and the cell suspension pipetted up and down for 2 min to lyse the cells. The suspension was then vortexed for 1 min to further homogenize the cell lysates and frozen at −80 °C.

Flow cytometry: Following their isolation, aliquots of the two white blood cell populations were stained with (1) a positive dye mix containing human CD2-FITC, human CD36-APC-Vio770, and human MC CD14 Monocyte Cocktail for staining CD2 and CD14 cells, respectively, and (2) a negative dye mix consisting of human CD45-VioBlue, mouse IgG2b-FITC, mouse IgG2a-PE, mouse IgM-APC, and mouse IgG2a-APC-Vio770 (Miltenyi Biotech). Only samples with purity of ≥90% for CD2 and CD14 were used in our study.

RNA extraction: RNA extraction was accomplished using the miRNeasy Mini Kit (Cat. No. 217004, Qiagen). In essence, the frozen CD2 and CD14 cell samples (−80 °C) were thawed in a 37 °C dry bath (~2.5 min) and incubated at room temperature for 5 min prior to the addition of 140 µL of chloroform and shaken vigorously for 15 s. Following a 3 min room temperature incubation, the samples were centrifuged at 12,000× *g* (4 °C, 15 min). The upper clear aqueous phase (~350 µL) was transferred to a 2 mL collection tube that was then placed inside the QIAcube (Cat. No. 9001292, Qiagen), and poly(A) RNA was extracted using the miRNeasy Mini Kit per manufacturer’s protocol. The quality and quantity of each RNA sample was determined on a Bioanalyzer 2100 (Agilent Technologies, Santa Clara, CA, USA). Finally, the RNA samples were frozen at −80 °C and shipped to the Yale Center for Genome Analysis (YCGA) (West Haven, CT) for RNA sequencing. Only samples with high RIN (RNA Integrity Number) ≥ 9 were sequenced.

RNA sequencing library preparation: Samples were sent to the Yale Center for Genome Analysis (YCGA; West Haven, CT, USA) for whole transcriptome RNA sequencing. mRNA was purified from approximately 200 ng of total RNA with oligo-dT beads and sheared by incubation at 94 °C. Following first-strand synthesis with random primers, second strand synthesis was performed with dUTP for generating strand-specific sequencing libraries. The cDNA library was then end-repaired, A-tailed, the adapters were ligated, and second-strand digestion was performed by uracil-DNA-glycosylase. Indexed libraries that met appropriate cut-offs for both were then quantified by qRT-PCR using a commercially available kit (KAPA Biosystems) and insert size distribution determined with the LabChip GX or Agilent Bioanalyzer. Samples with a yield of ≥0.5 ng/µL were sequenced.

Flow cell preparation and sequencing: Sample concentrations were normalized to 10 nM and loaded onto Illumina Rapid or high-output flow cells at a concentration that yields 130–250 million passing filter clusters per lane. Samples were sequenced using 75 bp paired-end sequencing on an Illumina HiSeq 2500 according to Illumina’s protocols. The 6 bp index is read during an additional sequencing read that automatically follows the completion of read 1. Data generated during sequencing runs were simultaneously transferred to the YCGA high-performance computing cluster. A positive control (prepared bacteriophage Phi X library) provided by Illumina is spiked into every lane at a concentration of 0.3% to monitor sequencing quality in real time.

Sequencing data processing: Signal intensities were converted to individual base calls during using the system’s Real Time Analysis (RTA) software. Sample demultiplexing was performed using Illumina’s CASAVA 1.8.2 software suite. Only data with sample error rate < 2% and a distribution of reads per sample in a lane that is within reasonable tolerance was used. Demultiplexed raw (FASTQ) RNA sequencing data was processed using Trimmomatic [39] for adaptor trimming, Bowtie2 [40] for alignment to the UCSC (University of California, Santa Cruz) hg19 transcriptome, and Express [41] for quantification. Processed reads yielded counts for 23,368 transcripts (gene symbols), corresponding to 29.8 ± 7.5 million and 33.9 ± 7.5 million mapped reads for CD2 and CD14 samples, respectively. Sample normalization to account for RNA concentration differences was performed using trimmed mean M-value (TMM) normalization [42]. The gene expression counts from each gene were determined for both CD2 and CD14 enriched cells separately. For each gene the ratio is determined as log(CD14/CD2), which is mathematically identical to log(CD14)−log(CD2).

Statistical analyses: Non-normal continuous clinical covariates, namely, total prostate specific antigen (PSA), prostate volume, and PSA density (PSAD) were log-transformed before further analyses. Models were built using a two-step procedure consisting of unsupervised variance-based transcript down-selection and classification by gradient boosting tree-based model (LightGBM) [43]. These models are then used to make predictions on the validation set.

Models were developed on the discovery set for each cell type alone (CD2 and CD14), and for the ratio (CD14/CD2). These are considered the genomic expression only models. The performance of various clinical data (age, race, DRE, family history, PSA and PSA density (PSAD which is PSA/prostate volume)) was evaluated and then combined with the ratio of CD14/CD2 to investigate possible enhancements to model performance. 

Model performance was evaluated by determining the area under the curve (AUC) of the receiver operating characteristic (ROC) curve [44]. Differences in performance are assessed using the DeLong test [45]. All analyses were performed in R, including the gene ontology and pathway associations using the enrichR package [46,47,48].

## 3. Results

Clinical and demographic characteristics of the independent training and validation sets are presented in Table 1. The entire cohort was collected in chronological order with the first 713 men serving as the discovery and training set, and last 315 men enrolled in the study being part of the independent validation set. While some small differences between patients in the training and validation sets were observed for age, race, and DRE, these do not have relevant clinical implications. Patients in the training set were slightly older, somewhat more likely to be Caucasian, and had fewer abnormal DREs. Differences in recruitment rates from the three different sites over the duration of the study most likely explain these minor differences observed in the clinicodemographic characteristics. No significant differences were observed for family history of prostate cancer, prostate volume, total PSA, PSAD, number of cancer-positive cores, maximum % of tumor involvement in a core, GG, or the adverse pathology binary endpoint. Missing data include 9.6% of prostate volume and 1.2% of PSA values leading to an overall 10.8% of cases where PSA density could not be calculated (Supplementary Figure S1). The worst pathology at any time during follow-up was used to define the patients’ status, in particular for the binary, adverse pathology endpoint, with a median follow-up of 3.8 years (interquartile range: 1.2–4.7 years) for the patients enrolled in this study.

Initial filtering of the transcriptomic data resulted in a reduced set of 18,703 transcripts with observed expression (nonzero counts) in at least 15% of the samples in either CD2 or CD14. While log-transformed CD14 and CD2 counts were analyzed separately, the CD2-normalized CD14 signal was also used as model input. The normalization consists of subtracting the log-transformed CD2 counts from the log-transformed CD14 counts per patient, on a gene-by-gene basis. This is also referred to as the log(CD14/CD2) ratio. Exploratory principal component analysis on log-transformed ratios of CD2 and CD14 data, log(CD14/CD2), revealed no significant batch effects between the training and validation datasets (Supplementary Figure S4).

The clinico-genomic model is built on a two-step procedure consisting of transcript down-selection and classification. For the former, we apply (unsupervised) variance-based transcript selection using the training set (*n* = 713). This down-selection stage is necessary to prevent the model from overfitting because the number of transcripts (17,138) is much larger than the number of subjects (*n* = 713), which is a well-recognized issue in regularized models when the effect sizes of individual transcripts are small. In the second step, we consider age, log-transformed total PSA, log-transformed PSAD, and the transcripts selected by variance as inputs to the model. To optimize the hyperparameters, namely, the number of transcripts selected by variance and the regularization parameter of the gradient boosting tree-based model used as classifier (LightGBM) [43] model, we use 10-fold cross-validation on the training set. Parameters of LightGBM other than the regularization strength were set to their default values. LightGBM is a gradient boosting machine (GBM) algorithm that combines (ensembles) the predictions of a collection of decision tress, each of which, considers a subset of model inputs, thus often resulting in performance improvements relative to standard approaches such as logistic regression. We verified that small variations of these hyperparameters did not substantially change the performance of the model. The final model is built on the entire training set restricted to the selected transcripts and the optimal regularization parameter found by cross-validation. This model is then used to make predictions on the validation set. 

**Figure 1:**
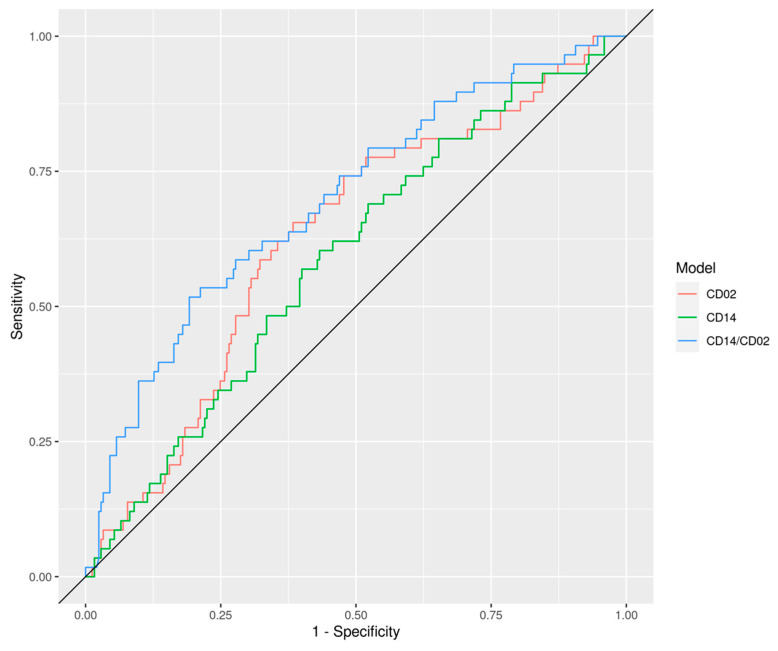
ROC curves for genomics only CD2, CD14, and CD14/CD2 ratio models. AUC values and confidence intervals are shown in the white area of Table 2.

Using this two-step procedure, transcriptomic models were built based on either the individual cell type counts for CD2 and CD14, but also using the CD14/CD2 ratio (Table 2). The genes and weighting factors of these models are shown in Supplementary Figure S2. The data demonstrates that the best performing immunotranscriptomic model is the one based on the CD14/CD2 ratio, emphasizing both the tumor phagocytosis mechanism and the anti-tumor immune response, yielded an AUC of 0.70. This compares favorably to the CD14 and CD2 only models, which resulted in AUCs of 0.59 (*p* = 0.033) and 0.63 (*p* = 0.079), respectively, indicating that the ratio has significantly increased performance in predicting adverse pathology over either cell type alone (Figure 1). Note that since the ratio (i) is taken on a log scale it is considered as a form of subtraction of underlying background within the immune system (reducing noise), and (ii) outperformed both individual CD14 and CD2 modalities, it was used as the basic immunotranscriptomic component for more advanced modeling exercises.

**Figure 2:**
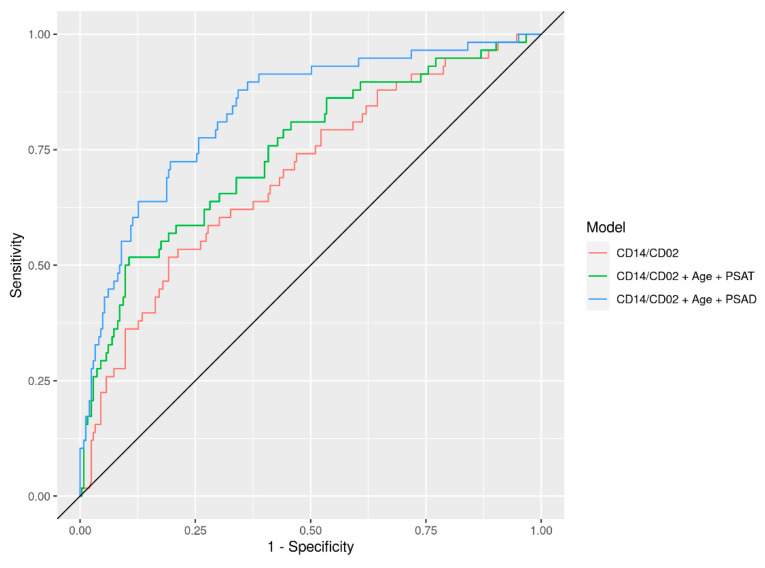
ROC curves for CD14/CD2 ratio model compared to those models including age, PSA, and PSAD. AUC values and confidence intervals are shown in Table 2 above.

The same LightGBM optimization procedure was also applied to combine immunotranscriptomics with readily available clinical and demographic risk factors. This resulted in increased performance in predicting adverse pathology compared to immunotranscriptomics alone. An immunotranscriptomics model combined with the simplest clinical risk factors, i.e., PSA and age, significantly (*p* = 0.02) outperformed these same clinical risk factors alone. While a measurement of prostate volume is not always readily available, it is known to be a significant risk factor in detecting adverse pathology and a good aid in the management of prostate cancer patients. When available, prostate volume is typically used to normalize PSA levels, resulting in a metric called PSAD (PSA/prostate volume). A model combining immunotranscriptomics with PSAD and age reached an AUC of 0.83 in the independent validation set (Figure 2), a significant improvement compared to a model based on age and PSA density alone, which yielded an AUC of 0.78 (*p* = 0.01). 

**Figure 3:**
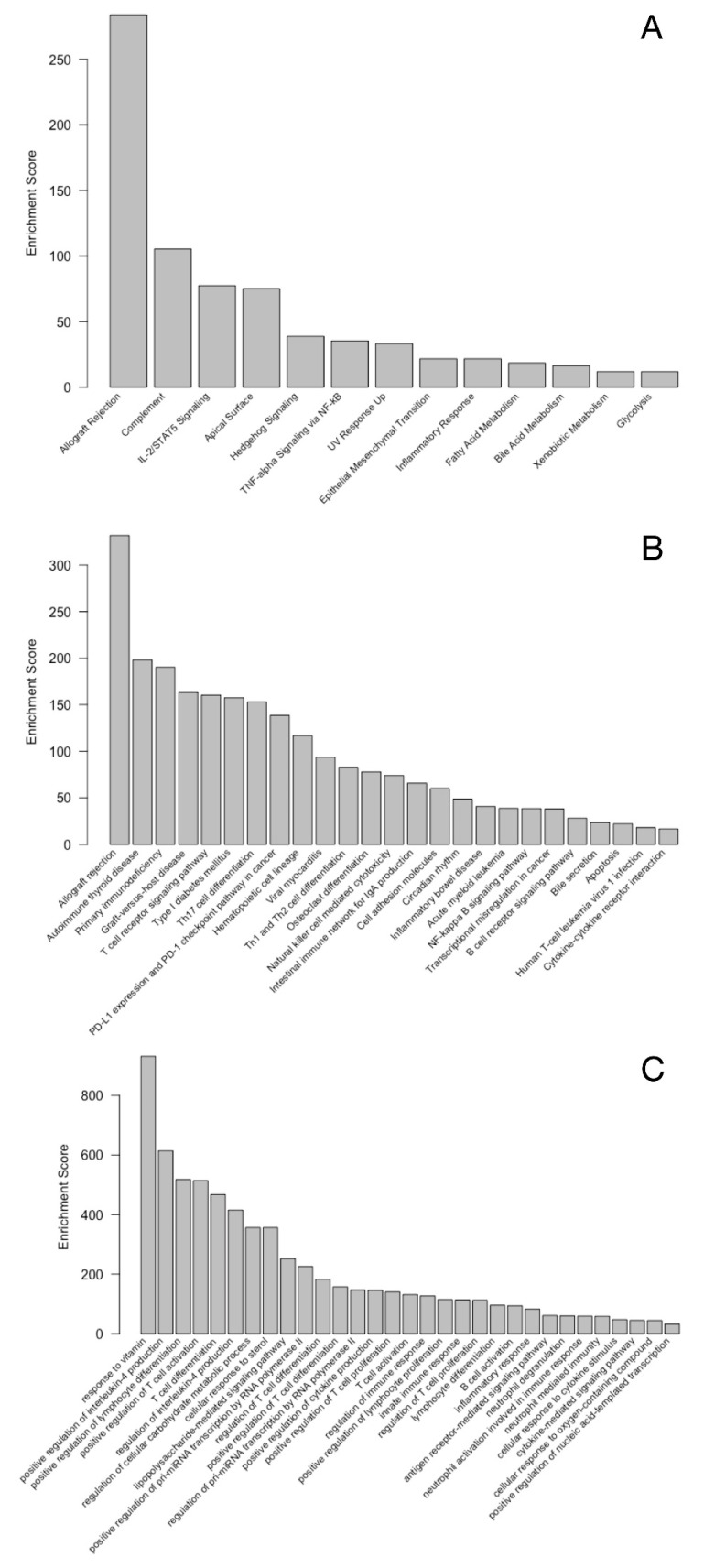
Top-ranked, enriched pathways and ontologies represented by the 120 genes in the best performing model according to MSigDB hallmark (**A**), KEGG (**B**), and gene ontology biological processes (**C**). Only terms that had a false discovery rate < 0.1 (or <0.01 for gene ontology (**C**)) are shown.

The biological relevance of the genes involved in the best performing model, i.e., immune-transcriptomics, age, and PSAD, was further evaluated through pathway and ontology analysis. This model included a set of 120 genes (Supplementary Table S1). As expected, immune response and immune system related pathways are significantly enriched (false discovery rate < 0.1) within this set of genes, as evidenced in the hallmarks MSigDB database (Figure 3A), KEGG database (Figure 3B), and gene ontology biological processes (Figure 3C). Interestingly, several cancer-related pathways also appear to be significantly over-represented by this geneset, most notably, hedgehog signaling (A), epithelial mesenchymal transition (A), PDL1 and PD1 checkpoint (B), and transcriptional misregulation in cancer (B). Other pathways can be linked to either the general function, response, or activation of phagocytic immune cells, or the presence of cancer cells, e.g., TNF alpha and NF-kappa B signaling, acute myeloid leukemia, and apoptosis. However, due to the nature of the gene selection procedure with the gradient boosting method, selecting for independent contributors, significant enrichment of certain pathways was not necessarily expected. Indeed, the set of 120 genes clearly link to phagocytic activity and cancer-related genes in KEGG. The phagocytic component is clearly represented by Fc gamma R-mediated phagocytosis and phagosome. In addition to the general pathways in cancer, choline metabolism in cancer, colorectal/breast/gastric cancer, microRNAs in cancer, proteoglycans in cancer appear in the list of KEGG-terms associated with the set of 120 genes included in the final model (Figure 3). 

To further explore the effect of patient age on the model performance, the analysis was also done by stratifying into age groups. This did not show statistically significant differences for most age group dependent models. The model including PSAD and age together with immunotranscriptomics showed a trend toward better performance in the 60–66-year age range with an AUC of 0.91 in the independent validation set, compared to the 42–60-year age range with an AUC of 0.79 (*p* = 0.06), and a significant difference in the 66–87 age range with an AUC of 0.80 (*p* = 0.04). Similar analysis was performed for race and DRE with results presented in Supplementary Table S2 which did not show significant differences; however, a trend was observed for race but the sample size was too small to draw definite conclusions. 

## 4. Discussion

The immune-transcriptomic profiling of purified populations of CD14+ monocytes and CD2+ lymphocytes by next-generation RNA sequencing provides a unique look into the pathways that are up or down regulated in patients with aggressive prostate cancer as defined by adverse pathologic features compared to biopsy negative controls and men with indolent pathologic disease. Since historic data sets with purified populations of immune cells are not available, adverse pathologic features were used a surrogate endpoint. These findings will need to be validated on clinically significant outcomes data once this data set matures and sufficient number of definitive events have occurred (metastasis and mortality). The specific genes in these pathways can be used in models predicting adverse prostate cancer pathology. Understanding the underlying biological phenomena and the cell types involved in this immunologic response to cancer provides insights, not only into the biologic pathways involved in the immune response to cancer, but also into potential novel biomarker strategies to manage cancer. It also demonstrates the systemic nature of the response that can be accessed via examining particular subpopulations of circulating immune cells.

Two distinct immunologic responses to early-stage prostate cancer are focused upon, the phagocytic and the immune response mechanisms, each harboring specific involvement in their response to an ongoing oncogenic process. Exploring this response as a ratio between these two cell type populations provides more information than exploring an individual cell type alone. This is likely due to the normalization effect (noise reduction) that using the ratio has on setting the baseline overall activity state of the immune system and showing the upregulation of multiple pathways including phagocytic and cancer pathways.

Future insights can be gained by looking at single cell RNA sequencing data to better understand the subsets of cells involved and how the proportions of these cell types shift with the development of cancer that escapes the immune surveillance system. This may allow for the deconvolution of bulk sequencing data on populations of circulating immune cells that are sequenced.

The gene sets and associated pathways uncovered by examining differential gene expression of circulating immune cells in the setting of early-stage prostate cancer highlights two different response mechanisms to early tumor development: (a) the tumor phagocytosis and (b) immune response mechanisms. Eventually, the genes associated with clinically significant cancer may also lead to identification of novel immune modulation therapeutic targets as well as markers for the development of prognostic and diagnostic models.

In conclusion, the novel clinico-immuno-genomic blood cell based approach utilizing gradient boosting described here demonstrates that (i) concurrent CD14+/CD2+ sequencing from the same patient is required to (a) filter out genomic signatures not associated with the disease, (b) achieve strong concordance with tissue biopsy testing results, and (c) substantially enhance the AUCs obtained from various current PCa clinical risk factors only, and (ii) the differential transcriptomic profiles of CD14+ and CD2+ cell populations are associated with and can predict adverse pathologic features of clinically localized prostate cancer. The performance of this strategy appears maximal in the peak years of prostate cancer detection. These results confirm the power of this novel technology, and further development should eventually aid in the management of PCa patients.

## Figures and Tables

**Table 1:** Cohort demographics for discovery and validation data sets. *p*-values are for significance testing for differences between the discovery and validation sets. Missing represents the percentage of cases where data was missing or unavailable.

	Level	Validation	Discovery	*p*	Missing
*n*		305	713		
age (mean (SD))		62.69 (7.60)	64.09 (7.89)	0.009	0
race (%)	AA	38 (12.5)	69 (9.7)	0.006	0
	Caucasian	227 (74.4)	593 (83.2)		
	Other	25 (8.2)	29 (4.1)		
	Unknown	15 (4.9)	22 (3.1)		
family history (%)	First	59 (19.3)	132 (18.5)	0.764	0
	None	204 (66.9)	494 (69.3)		
	Positive	7 (2.3)	12 (1.7)		
	Second	16 (5.2)	27 (3.8)		
	Unknown	19 (6.2)	48 (6.7)		
DRE (%)	Abnormal/Positive	74 (24.3)	114 (16.0)	0.002	0
	Normal/Negative (T1c)	203 (66.6)	550 (77.1)		
	Unknown	28 (9.2)	49 (6.9)		
volume (median (IQR))		40.20 (29.60, 54.08)	40.85 (30.78, 57.85)	0.264	9.6
psa_total (median (IQR))		4.97 (3.80, 7.10)	5.12 (3.80, 7.30)	0.3	1.2
psa_density (median (IQR))		0.12 (0.08, 0.19)	0.12 (0.07, 0.19)	0.929	10.8
cores_positive (median (IQR))		1.00 (0.00, 4.00)	1.00 (0.00, 4.00)	0.973	0
cores_percent (median (IQR))		4.00 (0.00, 30.00)	5.00 (0.00, 35.00)	0.862	0
site (%)	Unknown	0 (0.0)	55 (7.7)	<0.001	0
	CU	147 (48.2)	577 (80.9)		
	MIU	62 (20.3)	46 (6.5)		
	Urology Austin	96 (31.5)	35 (4.9)		
Gleason Group (%)	0	145 (47.5)	337 (47.3)	0.963	0
	1	32 (10.5)	78 (10.9)		
	2	66 (21.6)	166 (23.3)		
	3	38 (12.5)	77 (10.8)		
	4	13 ( 4.3)	27 ( 3.8)		
	5	11 (3.6)	28 (3.9)		
Adverse Pathology (%)	0	245 (80.9)	577 (80.9)	1	0.2
	1	58 (19.1)	136 (19.1)		

**Table 2:** ROC analysis results for various models showing discovery and validation AUC values with confidence intervals. Results are shown for discovery set (disc), and independent validation set (val). Colors indicate clinical variables used in models. Only validation AUC results by age tertial are shown—significance testing *p*-values are in Supplementary Figure S3. PSAT = total PSA and PSAD = PSA density. Clinical models are color coded with and without genomic component for ease of identifying the boost in performance achieved by adding genomics (CD14/CD2). Yellow (PSAT, Age), Blue (PSAT, Age, Race, DRE, FamH), and Orange (PSAD, Age).

Model	AUC (disc)	AUC (val)	AUC (val)		
			Age (42,60)	Age (60,66)	Age (66,87)
Age	0.60 (0.54, 0.65)	0.56 (0.47, 0.65)			
PSAT	0.72 (0.67, 0.77)	0.67 (0.59, 0.75)	0.63 (0.48, 0.77)	0.74 (0.59, 0.89)	0.66 (0.52, 0.82)
Vol	0.60 (0.55, 0.66)	0.72 (0.65, 0.80)	0.75 (0.65, 0.85)	0.82 (0.67, 0.96)	0.62 (0.48, 0.76)
PSAD	0.77 (0.72, 0.81)	0.78 (0.71, 0.85)	0.76 (0.64, 0.89)	0.88 (0.80, 0.97)	0.74 (0.61, 0.87)
Clinical (PSAT, Age)	0.72 (0.67, 0.77)	0.67 (0.59, 0.75)	0.60 (0.46, 0.75)	0.73 (0.58, 0.88)	0.67 (0.54, 0.80)
Clinical (PSAT, Age, Race, DRE, FamH)	0.73 (0.69, 0.78)	0.73 (0.65, 0.80)	0.70 (0.56, 0.83)	0.72 (0.56, 0.88)	0.72 (0.61, 0.83)
Clinical (PSAD, Age)	0.78 (0.74, 0.82)	0.78 (0.71, 0.85)	0.73 (0.61, 0.86)	0.89 (0.80, 0.97)	0.76 (0.64, 0.88)
CD2	0.80 (0.76, 0.84)	0.63 (0.55, 0.71)	0.67 (0.53, 0.80)	0.53 (0.35, 0.71)	0.61 (0.49, 0.74)
CD14	0.82 (0.77, 0.86)	0.59 (0.51, 0.67)	0.60 (0.46, 0.74)	0.54 (0.36, 0.73)	0.63 (0.51, 0.75)
CD14/CD2	0.72 (0.67, 0.77)	0.70 (0.62, 0.77)	0.76 (0.65, 0.88)	0.63 (0.47, 0.79)	0.68 (0.55, 0.81)
CD14/CD2 + Clinical (PSAT, Age)	0.99 (0.99, 1.00)	0.75 (0.67, 0.82)	0.73 (0.60, 0.86)	0.72 (0.57, 0.88)	0.76 (0.64, 0.88)
CD14/CD2 + Clinical (PSAT, Age, Race, DRE, FamH)	0.97 (0.95, 0.99)	0.76 (0.69, 0.83)	0.78 (0.66, 0.90)	0.72 (0.55, 0.89)	0.75 (0.63, 0.86)
CD14/CD2 + Clinical (PSAD, Age)	0.91 (0.88, 0.93)	0.83 (0.77, 0.89)	0.79 (0.67, 0.92)	0.91 (0.84, 0.97)	0.80 (0.70, 0.90)

## Data Availability

The RNA sequencing count data and adverse pathology endpoint presented in this study will be openly available in FigShare at: dx.doi.org/10.6084/m9.figshare.16602191.

## Supplementary Materials

The following are available online at https://www.mdpi.com/article/10.3390/cells10102567/s1, Figure S1: Data Integrity, Figure S2: Signatures–expression only, Figure S3: Significance tests, Figure S4: UMAP for XPR+age+PSA. Table S1: 120 genes from best performing model. Table S2 combined clinical and genomic model for race and DRE subgroups.
