# Supplementary material for: Evaluation of an RNAseq-Based Immunogenomic Liquid Biopsy Approach in Early-Stage Prostate Cancer
Source: Cells. 2021 Sep 28;10(10):2567. doi: 10.3390/cells10102567 (PMC8533765; doi:10.3390/cells10102567)
Supplement: Supplementary file 1 [file cells-10-02567-s001.zip › cells-1353123-SI.pdf]

Supplementary figure legends

Supplementary Figure S1 – Data Integrity

N=1018 (713 discovery, 305 validation)
PSA total missing: 12 (imputed with median, 5.1)
Vol missing: 99 (imputed with median, 40.8)
PSA density missing: 110 (imputed with median, 0.12)
Race missing: 37 (imputed with, Unknown)
FamH missing: 67 (imputed with, Unknown)
DRE missing: 77 (imputed with, Unknown)

Samples with missing PSA or Vol: 111

GG	0	1	2	3	4	5	Total
Validation	145	32	66	38	13	11	305
Discovery	337	78	166	77	27	28	713
Total	482	110	232	115	40	39	1018

Age tertiles	1	2	3
Discovery	[40,61]	(61,67]	(67,86]
	267	214	232
Validation	[42,60]	(60,66]	(66,87]
	119	95	91

Adverse Pathology	0	1	Total
Validation	245	58	305
Discovery	577	136	713
Total	822	194	1018

Figure S1: Data integrity parameters with missing data numbers and imputation strategy used.

Supplementart Figure S2 Signatures (Expression Only)

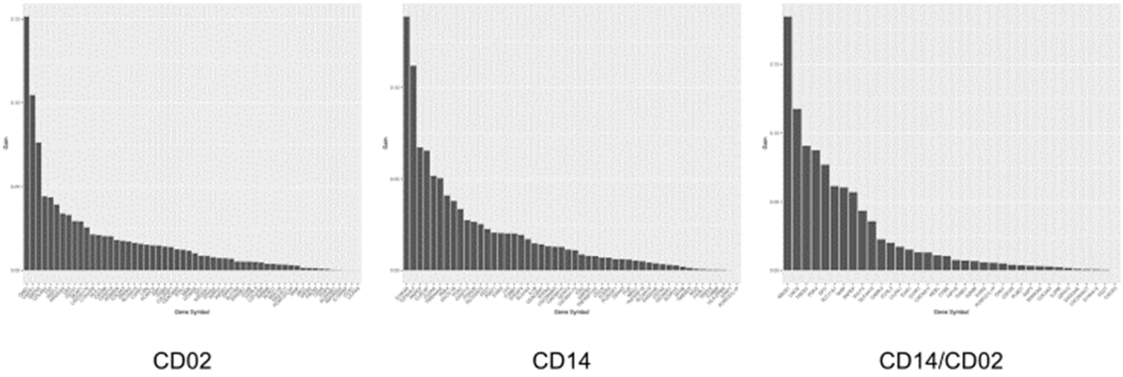

Figure S2: Three genomic expression only models with gene symbols and weightings (gains).

Supplementary Figure S3 - Significance Tests

Modality 1	Modality 2	p-Value
CD02 (All)	CD14/CD02 (All)	0.08
CD14 (All)	CD14/CD02 (All)	**0.03**
CD02 (Age G1)	CD14/CD02 (Age G1)	0.11
CD14 (Age G1)	CD14/CD02 (Age G1)	**0.04**
CD02 (Age G2)	CD14/CD02 (Age G2)	0.12
CD14 (Age G2)	CD14/CD02 (Age G2)	0.22
CD02 (Age G3)	CD14/CD02 (Age G3)	0.22
CD14 (Age G3)	CD14/CD02 (Age G3)	0.30

Modality 1 + Age + PSAT	Modality 2 + Age + PSAT	p-Value
CD02 (All)	CD14/CD02 (All)	0.41
CD14 (All)	CD14/CD02 (All)	**0.03**
CD02 (Age G1)	CD14/CD02 (Age G1)	0.30
CD14 (Age G1)	CD14/CD02 (Age G1)	**0.02**
CD02 (Age G2)	CD14/CD02 (Age G2)	0.92
CD14 (Age G2)	CD14/CD02 (Age G2)	0.89
CD02 (Age G3)	CD14/CD02 (Age G3)	0.06
CD14 (Age G3)	CD14/CD02 (Age G3)	0.07
Age	CD14/CD02 (All)	**0.0001**
PSAT	CD14/CD02 (All)	**0.02**

Modality 1 + Age + PSAD	Modality 2 + Age + PSAD	p-Value
CD02 (All)	CD14/CD02 (All)	0.31
CD14 (All)	CD14/CD02 (All)	**0.01**
CD02 (Age G1)	CD14/CD02 (Age G1)	0.35
CD14 (Age G1)	CD14/CD02 (Age G1)	**0.003**
CD02 (Age G2)	CD14/CD02 (Age G2)	0.08
CD14 (Age G2)	CD14/CD02 (Age G2)	0.74
CD02 (Age G3)	CD14/CD02 (Age G3)	0.42
CD14 (Age G3)	CD14/CD02 (Age G3)	0.08
CD14/CD02 (Age G1)	CD14/CD02 (Age G2)	0.06
CD14/CD02 (Age G3)	CD14/CD02 (Age G2)	**0.04**
Age	CD14/CD02 (All)	**5.1e-08**
PSAT	CD14/CD02 (All)	**2.1e-08**
PSAD	CD14/CD02 (All)	**0.01**

Figure S3: p-values for significance testing between modality 1 vs modality 2. Modalitites include cell types, the ratio of expression between cell types and clinical factors. G1, G2 and G3 represent the low [40,61], middle [61,67] and high [67,86] tertials of age.

Supplementary Figure S4 - UMAP for XPR+age+PSA

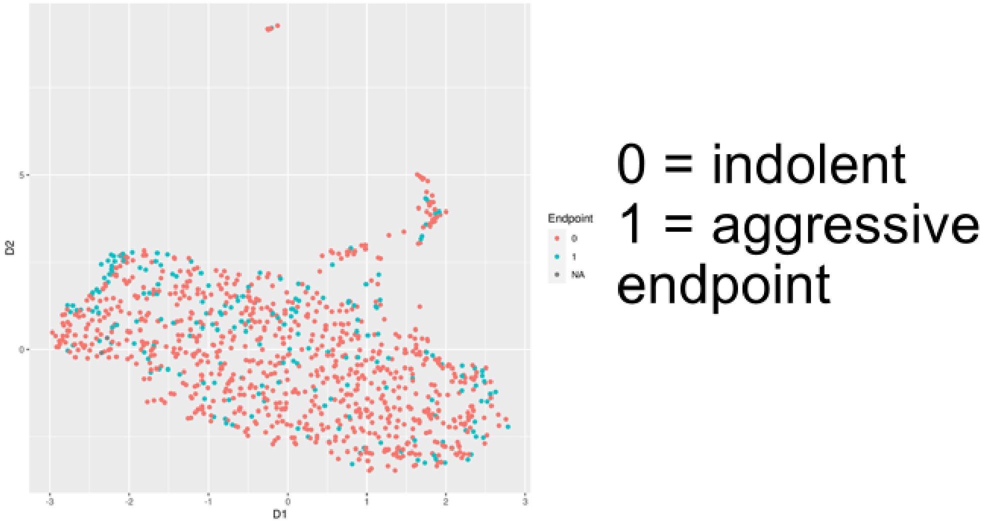

Figure S4: XPR = Genomic expression signature, UMAP = Uniform Manifold Approximation and Projection

Table S1

	mnames	symbol	Feature	Gain	Cover	Frequency
3	cd14/cd02	CACNA1I	2108	0.025765869	0.035050536	0.041189931
4	cd14/cd02	PDK4	10863	0.017543563	0.031015348	0.025171625
5	cd14/cd02	TREML4	15410	0.016720457	0.029176667	0.029748284
6	cd14/cd02	ODZ1	10457	0.015323098	0.020517253	0.022883295
7	cd14/cd02	COL5A3	3125	0.014934649	0.01399379	0.016018307
8	cd14/cd02	SLC11A1	13337	0.014919125	0.012909299	0.016018307
9	cd14/cd02	GZMM	5961	0.012267362	0.016889437	0.016018307
10	cd14/cd02	LOC283174	8022	0.011336678	0.022752296	0.016018307
11	cd14/cd02	KRT72	7266	0.011233644	0.016872922	0.016018307
12	cd14/cd02	SLC4A10	13561	0.010039807	0.025581882	0.029748284
13	cd14/cd02	LBH	7339	0.008867283	0.011890868	0.013729977
14	cd14/cd02	CHST15	2875	0.008530193	0.013168036	0.013729977
15	cd14/cd02	COL6A2	3127	0.008275952	0.017676656	0.018306636
16	cd14/cd02	ZNF683	16950	0.008198293	0.012375311	0.018306636
17	cd14/cd02	ALPL	471	0.007993247	0.012865259	0.016018307
18	cd14/cd02	CSF1R	3313	0.007650001	0.011995464	0.016018307
19	cd14/cd02	SCGB3A1	12932	0.006532129	0.013206571	0.011441648
20	cd14/cd02	POM121L1P	11401	0.006351518	0.013162531	0.013729977
21	cd14/cd02	SAMD3	12838	0.005979345	0.015595755	0.013729977
22	cd14/cd02	FAM198B	4727	0.005812442	0.005791293	0.006864989
23	cd14/cd02	NEBL	10013	0.005515268	0.013839649	0.011441648
24	cd14/cd02	RASGRP1	12144	0.005333506	0.009149363	0.006864989
25	cd14/cd02	KYNU	7284	0.005150843	0.012964349	0.013729977
26	cd14/cd02	RTN1	12763	0.005113733	0.018953824	0.016018307
27	cd14/cd02	CTSW	3398	0.004649075	0.00695836	0.006864989
28	cd14/cd02	RORC	12545	0.004534373	0.006523462	0.009153318
29	cd14/cd02	ABCB1	35	0.004300039	0.008422698	0.009153318
30	cd14/cd02	LMO2	7559	0.004273154	0.008714465	0.006864989
31	cd14/cd02	S100A12	12803	0.004083351	0.007850175	0.004576659
32	cd14/cd02	CD27	2499	0.004070016	0.00265893	0.004576659
33	cd14/cd02	SH2D1A	13195	0.004000255	0.010932993	0.011441648
34	cd14/cd02	PDZD4	10886	0.003794164	0.011263295	0.009153318
35	cd14/cd02	CD300LB	2508	0.003775868	0.003787462	0.006864989
36	cd14/cd02	FPR2	5247	0.003741399	0.00526281	0.004576659
37	cd14/cd02	CLEC4F	2970	0.003716222	0.00529584	0.006864989
38	cd14/cd02	AQP3	716	0.003545167	0.006600533	0.006864989
39	cd14/cd02	FOS	5211	0.003434624	0.003616806	0.006864989
40	cd14/cd02	PTCH1	11819	0.003296609	0.008758505	0.009153318
41	cd14/cd02	ASGR2	917	0.003279304	0.005884879	0.004576659
42	cd14/cd02	MARCKS	8736	0.003197114	0.00435448	0.004576659
43	cd14/cd02	SIGLEC14	13270	0.003107705	0.003941603	0.006864989
44	cd14/cd02	EGR1	4209	0.003056205	0.009589765	0.011441648

45	cd14/cd02	MPEG1	9468	0.002911722	0.003303019	0.004576659
46	cd14/cd02	SYNE2	14488	0.002881955	0.004943518	0.004576659
47	cd14/cd02	POM121L4P	11402	0.002781306	0.001073481	0.00228833
48	cd14/cd02	CD40LG	2524	0.002733438	0.002587365	0.00228833
49	cd14/cd02	PYGL	11944	0.002557105	0.003363574	0.004576659
50	cd14/cd02	MYCL1	9748	0.002481153	0.002587365	0.00228833
51	cd14/cd02	CSMD1	3320	0.002458057	0.00093035	0.00228833
52	cd14/cd02	BACH2	1148	0.002421843	0.002003832	0.004576659
53	cd14/cd02	ENO2	4354	0.002407792	0.00442054	0.004576659
54	cd14/cd02	LOXHD1	8389	0.002392796	0.002224033	0.00228833
55	cd14/cd02	LILRA1	7426	0.002156558	0.004459076	0.006864989
56	cd14/cd02	SLC44A5	13550	0.002155522	0.002598375	0.004576659
57	cd14/cd02	CCDC64	2360	0.002108029	0.003837007	0.006864989
58	cd14/cd02	IGSF9B	6563	0.002051396	0.00179464	0.00228833
59	cd14/cd02	RORA	12543	0.00204954	0.001607469	0.00228833
60	cd14/cd02	CD6	2536	0.002025291	0.006831744	0.009153318
61	cd14/cd02	SCML4	12940	0.001997796	0.002642415	0.00228833
62	cd14/cd02	RHOH	12375	0.001962694	0.005400436	0.006864989
63	cd14/cd02	VCAN	16018	0.001944135	0.000660604	0.00228833
64	cd14/cd02	CXCR3	3443	0.001938351	0.011775263	0.006864989
65	cd14/cd02	PID1	11055	0.001898757	0.009215423	0.006864989
66	cd14/cd02	EGR2	4210	0.001772491	0.005637152	0.006864989
67	cd14/cd02	PRF1	11607	0.001745359	0.003143373	0.004576659
68	cd14/cd02	NFAM1	10062	0.001724394	0.008841081	0.006864989
69	cd14/cd02	SAPCD2	12854	0.001711176	0.004315945	0.004576659
70	cd14/cd02	ADAMTSL4	228	0.001689127	0.003704886	0.006864989
71	cd14/cd02	TARP	14583	0.00162089	0.003897562	0.006864989
72	cd14/cd02	PYHIN1	11947	0.001613049	0.000946865	0.00228833
73	cd14/cd02	SKAP1	13310	0.00156263	0.004756347	0.004576659
74	cd14/cd02	SPI1	14129	0.001538732	0.001409288	0.00228833
75	cd14/cd02	MAL	8630	0.001512475	0.002273578	0.00228833
76	cd14/cd02	KLRB1	7225	0.001475973	0.002400194	0.00228833
77	cd14/cd02	KRT1	7257	0.001434266	0.002791051	0.006864989
78	cd14/cd02	B3GAT1	1118	0.00143112	0.003336049	0.006864989
79	cd14/cd02	ANPEP	613	0.001378165	0.003925088	0.00228833
80	cd14/cd02	CD86	2551	0.001240784	0.001205602	0.00228833
81	cd14/cd02	MS4A14	9597	0.001223076	0.008169467	0.006864989
82	cd14/cd02	FGFBP2	5039	0.001218224	0.003016757	0.00228833
83	cd14/cd02	MS4A7	9602	0.001213586	0.006204171	0.004576659
84	cd14/cd02	GPR15	5754	0.001088714	0.003319534	0.006864989
85	cd14/cd02	CCR4	2447	0.000999904	0.001431308	0.00228833
86	cd14/cd02	F13A1	4554	0.000965831	0.003925088	0.00228833
87	cd14/cd02	CD247	2497	0.000932436	0.002620395	0.00228833
88	cd14/cd02	EOMES	4377	0.00092602	0.001800145	0.00228833
89	cd14/cd02	CD1D	2485	0.000907206	0.004651752	0.006864989

90	cd14/cd02	C19orf59	1677	0.000884088	0.001860701	0.00228833
91	cd14/cd02	LOC284837	8060	0.000868144	0.002702971	0.00228833
92	cd14/cd02	TC2N	14676	0.000827999	0.002422214	0.00228833
93	cd14/cd02	LCK	7345	0.000793051	0.00704644	0.006864989
94	cd14/cd02	GZMB	5958	0.000733918	0.000759694	0.00228833
95	cd14/cd02	GZMA	5957	0.000731485	0.00429943	0.004576659
96	cd14/cd02	NAPSB	9872	0.000677812	0.003187413	0.004576659
97	cd14/cd02	ZAP70	16363	0.000651416	0.00264792	0.004576659
98	cd14/cd02	ALDH1A1	421	0.000618264	0.001260652	0.00228833
99	cd14/cd02	CD28	2502	0.000596694	0.001811155	0.00228833
100	cd14/cd02	HIP1R	6154	0.000567496	0.0008753	0.00228833
101	cd14/cd02	GPR56	5783	0.000528153	0.003837007	0.00228833
102	cd14/cd02	S100A9	12811	0.000492099	0.000451413	0.00228833
103	cd14/cd02	CLEC12A	2953	0.000482809	0.004618722	0.004576659
104	cd14/cd02	GATA3	5405	0.000441024	0.001260652	0.00228833
105	cd14/cd02	CD5	2529	0.000414809	0.001332218	0.00228833
106	cd14/cd02	CST7	3342	0.000377496	0.000346817	0.00228833
107	cd14/cd02	CLEC4E	2969	0.000337332	0.002113932	0.00228833
108	cd14/cd02	TIGIT	14883	0.000278518	0.003187413	0.004576659
109	cd14/cd02	HCK	6039	0.000265714	0.002653425	0.00228833
110	cd14/cd02	ATP1A4	1003	0.000243019	0.002521304	0.00228833
111	cd14/cd02	AQP9	720	0.000233741	0.001040451	0.00228833
112	cd14/cd02	GZMH	5959	0.000206169	0.00093035	0.00228833
113	cd14/cd02	SULF2	14420	0.000147204	0.002058882	0.00228833
114	cd14/cd02	LILRA3	7428	0.000139055	0.000974391	0.00228833
115	cd14/cd02	CD14	2472	9.39E-05	0.000407372	0.00228833
116	cd14/cd02	IL7R	6627	7.09E-05	0.000363332	0.00228833
117	cd14/cd02	RIN2	12401	4.08E-05	0.002950697	0.00228833
118	cd14/cd02	VNN1	16051	3.37E-05	0.001156057	0.004576659
119	cd14/cd02	NCALD	9908	2.78E-05	0.00088631	0.00228833
120	cd14/cd02	TREM1	15406	2.75E-05	0.000478938	0.00228833
121	cd14/cd02	SYCP2L	14476	7.65E-06	0.00171757	0.00228833
122	cd14/cd02	LGALS2	7399	5.01E-06	0.000600048	0.00228833

Statistical modeling assumptions:

1. The discovery and validation cohorts are independently and identically distributed samples from the same population.
2. The ranking of the gene-wise variances of gene expression data between discovery and validation cohorts are consistent.

Table S2: AA = African American, DRE = digital rectal exam. CD14/CD2 is genomics only model performance while CD14/CD2 + Clinical (PSAD, Age) is combined clinical and genomic model. PSAD = PSA density i.e. PSA/prostate gland volume.

**Model**	**AUC (val)**		p value
	Race (not AA)	Race (AA)	
CD14/CD2 + Clinical (PSAD, Age)	0.85 (0.78, 0.91)	0.72 (0.55, 0.89)	0.09
CD14/CD2	0.69 (0.61, 0.78)	0.67 (0.44, 0.90)	0.42
	DRE (normal)	DRE (abnormal)	
CD14/CD2 + Clinical (PSAD, Age)	0.84 (0.77, 0.91)	0.82 (0.71, 0.93)	0.36
CD14/CD2	0.72 (0.62, 0.82)	0.64 (0.50, 0.78)	0.18
